# Solvent type influences bond strength to air or blot-dried dentin

**DOI:** 10.1186/s12903-016-0247-3

**Published:** 2016-08-22

**Authors:** Özgür Irmak, İsmail Hakkı Baltacıoğlu, Nuran Ulusoy, Yıldırım Hakan Bağış

**Affiliations:** 1 Department of Restorative Dentistry, Faculty of Dentistry, Eskişehir Osmangazi University, 26480 Eskişehir, Turkey; 2 Department of Restorative Dentistry, Faculty of Dentistry, Ankara University, 06500 Ankara, Turkey; 3 Department of Restorative Dentistry, Faculty of Dentistry, Near East University, Near East Boulevard, ZIP: 99138 Nicosia/TRNC Mersin 10, Turkey

**Keywords:** Solvent, Adhesive, Shear bond strength, Tertiary butanol, Blot drying

## Abstract

**Background:**

Air-drying of etched and rinsed dentin surface may force the exposed collagen fibrils to collapse. Blot-drying is an alternative method to wipe the excess water from the dentin surface without compromising the monomer penetration. Contemporary total etch adhesives contain ethanol/water or acetone as solvent in which resin monomers are dissolved. Solvent type of the adhesive system has an important role in bonding to dentin. An adhesive containing tertiary butanol as an alternative solvent has been in the market. Purpose of this study is to determine the shear bond strengths of three total-etch adhesives with different solvents (acetone, ethanol or tertiary butanol) applied to air or blot dried moist dentin.

**Methods:**

Sixty extracted non-carious human third molars were divided into three main groups according to solvent content of the adhesives [acetone based - One Step (OS, Bisco, IL, USA); ethanol/water based - Optibond Solo Plus (OB, Kerr, CA, USA); and tertiary butanol based - XP Bond (XP, Caulk/Dentsply, DE, USA)]. Each main group was divided into two groups according to drying methods (blot or air) (*n* = 10). Shear bond strengths (SBS) were measured. Data were analyzed by Student’s *t* test and Tukey HSD test (*p* < 0,05).

**Results:**

XP showed highest SBS values in both drying methods applied (*p* < 0.05). Drying method did not influence the SBS in OS and OB (*p* > 0.05). XP-blot produced significantly higher SBS than XP-air (*p* < 0.05).

**Conclusions:**

Tertiary butanol based adhesive showed higher bond strength values than ethanol or acetone based adhesives. Blot drying of dentin improved the bond strength values of tertiary butanol based adhesive. Further research is necessary to determine in vivo and in vitro performance of tertiary butanol based adhesives.

## Background

Establishing an effective bond between dental substrates and resin composite still remains a challenge in restorative dentistry. Type of the adhesive system has an important role in bonding to dentin and has an influence on the clinical performance of the resin composite restoration [1]. In two-step total-etching technique, dentin surface is conditioned with phosphoric acid which demineralizes inorganic content of dentin to some depth and leaves the collagen fibrils exposed [2]. After etching, dentin surface is rinsed with water. Finally, adhesive is applied and light cured. However, before the application of adhesive, excess water should be removed from the dentin surface. It is recommended that the dentin surface be kept moist for better monomer penetration [3]. This concept is known as “wet-bonding” technique, in which, dentin is dried and left visibly moist. Generally, air stream is used for this purpose. While applying air to the dentin surface, water evaporates from the exposed collagen of demineralized dentin. This evaporation forces the exposed collagen fibrils to collapse [4], thus reduces interfibrillar spaces and decreases the monomer penetration [5]. To prevent this collapse of collagen fibrils, an alternative method was suggested to dry the acid-etched dentin surface, which is known as “blot-drying” [6]. In this technique, etched dentin surface is wiped with a tissue or a sponge, leaving the surface visibly moist.

Contemporary total-etch (TE) adhesives mainly contain ethanol/water or acetone as a solvent, in which resin monomers are dissolved [7]. Solvents are responsible for water displacement from collagen network and infiltration of resin monomers into spaces previously occupied by water [6]. Composition of the adhesive and solvent type requires different moisture spectrums [8, 9]. Acetone based systems evaporate much residual water than ethanol/water based systems; however, they are more sensitive to air-drying as they cannot re-expand the shrunken collagen fibrils [10]. Ethanol/water based systems are less moisture sensitive and good at re-expanding collagen matrix and produce higher bond strengths in dried dentin [11]. Wet bonding may be the ideal technique for current adhesives; however this moisture concept varies widely among clinicians and manufacturers. Drying time and air-syringe distance, air pressure are variables that also have an effect on bond strengths and not easy to control [12].

An adhesive containing a different solvent, tertiary butanol is on the market. According to its manufacturer, this adhesive system has a high degree of technique robustness due to chemical composition of tertiary butanol (XP BOND. Konstanz, Germany: Dentsply DeTrey, 2006). In literature there are not many studies investigating the effect of tertiary butanol containing TE adhesive on bond strength to dentin. Additionally, information about the bonding performance of this solvent on air or blot-dried dentin is scarce. Therefore, this study aimed to evaluate the effects of organic solvents (acetone, ethanol or tertiary butanol) on shear bond strength (SBS) of three different TE adhesive systems, applied after dentin is air or blot-dried. The null hypotheses tested were: 1) bond strength would not be dependent on drying skills applied to dentin; 2) type of solvent would not have an influence on bond strength to dentin.

## Methods

**Fig. 1:**
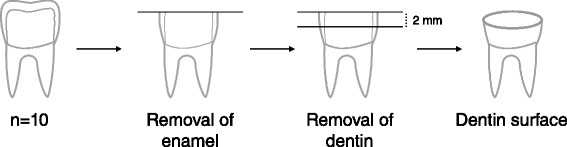
Dentin specimen preparation

**Fig. 2:**
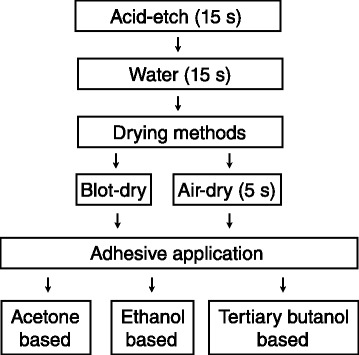
Experimental design of the study

Sixty extracted non-carious human third molars with similar crown heights were used in the study. Informed consents and ethical approval were obtained from Ankara University Faculty of Dentistry Ethical Committee. Teeth were kept in 0,1 % thymol at 4 °C for no longer than 2 weeks. A written informed consent was obtained from all patients. Teeth were embedded in epoxy resin. A model trimmer was used to grind away only occlusal enamel, exposing a flat superficial dentin surface (Fig. 1). This superficial dentin was used as a reference plane for further preparation. Deep dentin surface was obtained by cutting 2 mm below the reference plane using a low speed saw (Micracut 175, Metkon Instruments Ltd, Bursa, Turkey) under running water. Specimens that showed any visible pulp exposures were excluded from the study. Each dentin surface was then polished under running water by 600-grit SiC papers for 30 s to create a smear layer. Three total-etch adhesive systems with three different solvents were tested: One Step (OS) acetone-based; Optibond Solo Plus (OB) ethanol-/water based; and XP Bond (XP) tertiary butanol based systems. Table 1 shows the mode of application, components and manufacturers of these adhesives. Figure 2 shows experimental design of the study.

**Table 1:** Mode of application, compositions and manufacturers of tested adhesives

Materials	Components	Mode of application	Manufacturer	Lot
One Step (OS)	Bis-GMA, HEMA, BPDM, initiator and acetone	Apply a minimum of two generous coats, Agitate slightly for 10–15 s, gently air dry to evaporate solvent for 5 s, light cure for 10 s	Bisco, Schaumburg IL, USA	0700003510
Optibond Solo Plus (OB)	Bis-GMA, HEMA, GPDM, sodium-fluorsilicate, ethanol, water, initiator	Apply surface with applicator tip for 15 s using light brushing motion, air thin for 5 s, light cure for 20 s	Kerr Corporation, Orange, CA, USA	08639
XP Bond (XP)	PENTA, TCB resin, UDMA, TEGDMA, HEMA, nanofiller, initiators, butylated benzenediol, tertiary butanol	Wet all cavity surfaces uniformly, leave the surface undisturbed for 20 s, evaporate solvent for at least 5 s, light cure for a minimum of 10 s.	Caulk/Dentsply, Milford, DE, USA	0703002776

Teeth were randomly divided into 3 main groups for adhesives (acetone, ethanol or tertiary butanol based) and 2 sub-groups for drying conditions (blot or air). In each group, entire dentin surface was etched with 37 % phosphoric acid gel (Super Etch, SDI Inc, Bensenville IL, USA) for 15 s and rinsed with distilled water for 15 s. In blot-drying groups, a foam pellet (Foam Pellet, Bisco, Inc., USA) was used to wipe off excess water, leaving the surface visibly moist (blot). In air-drying groups, dentin surfaces were dried with gentle stream of oil-free compressed air for 5 s, from 10 cm distance, with an horizontal angle of 45° to remove excess water, leaving the surface visibly moist (air). Adhesive systems were applied according to the manufacturers’ instructions and light-cured with halogen curing light (Hilux Ultra, Benlioğlu Dental, Ankara, Turkey) with an intensity of at least 800 mW/cm^2^. Light intensity was checked before each curing session with the radiometer of the curing unit. After adhesive application, cylinders of composite resin (Clearfil Majesty Esthetic A2, Kuraray, Osaka, Japan, LOT # 00004B) were bonded by using a teflon mold with inner diameter of 3,6 mm. Composite build-ups were constructed in double 2 mm increments with each increment being cured for 20 s. All adhesive procedures were performed by a single operator. Specimens were then stored in distilled water at 37 °C for 24 h prior to SBS testing. Universal testing machine (LRX, Lloyd Instruments, UK) was used to measure the SBS of the adhesive systems. A notched flat guillotine blade was attached to the coupler in horizontal position. The guillotine was able to glide up and down and engage to the composite stub on the bonded surface passively between the dentin-resin interface. SBS test was performed by a single, trained operator, at a crosshead speed of 0.5 mm/min. The SBS in terms of MPa was calculated. Data were analyzed by student’s *t*-test and Tukey HSD test at a probability level of 0.05 (SPSS 15.0, SPSS Inc., Chicago, IL, USA). After SBS testing, specimens were examined with a stereomicroscope at 4.5-x magnification (Olympus ZS 61, Olympus Corp.; Tokyo, Japan) to determine the mode of fracture. Failure modes were classified as adhesive, cohesive dentin, cohesive composite and mixed.

## Results

Mean shear bond strengths for each group are presented in Table 2. XP-air produced significantly higher SBS values than OS-air (*p* = 0.036) and OB-air (*p* = 0.005). Similarly, XP-blot produced significantly higher SBS values than OS-blot (*p* = 0.034) and OB-blot (*p* = 0.000). When comparing drying methods there was no significant difference in OS (*p* = 0.061) and OB (*p* = 0.441). However, in XP, there was a significant difference, as XP-blot produced significantly higher SBS than XP-air (*p* = 0.040). There were no cohesive failures in tested specimens. Majority of failure patterns were adhesive for OS and OB in both drying methods. XP showed both adhesive and mixed failure patterns in both drying methods.

**Table 2:** Mean ± standard deviation (MPa) of shear bond strengths as a function of drying method

	OS	OB	XP
Air	13.82 ± 5.22^Aa^	12.09 ± 4.59^Aa^	19.40 ± 4.36^Ba^
Blot	18.44 ± 5.11^Aa^	13.57 ± 3.81^Aa^	23.75 ± 4.42^Bb^

Means sharing a letter are not significantly different (*p* > 0.05)

Upper cases compare data in the same row and lower cases in the same column

## Discussion

The first null hypothesis was rejected; as air-drying resulted in lower bond strength than blot-drying for all groups. This difference was significant between XP-air and XP-blot (*p* = 0.040). The second null hypothesis that the type of solvent would not have an influence on bond strength to dentin had also to be rejected; as tertiary butanol containing adhesive system was found to significantly improve SBS to dentin for both drying methods.

In order to obtain a good penetration of adhesives into collagen network it is important to keep interfibrillar spaces stiff when drying the etched dentin surface. If dentin surface is excessively air-dried collagen network collapses. As the collagen shrinks, water in tubules is trapped and there is no interaction with the primer and/or adhesive [6]. This results in reduced adhesive penetration and poor bond strength. Besides, if the etched dentin surface is overwet then collagen network is swelled and interfibrillar spaces decrease [13]. Therefore, it may not be easy to keep the etched dentin surface in an optimum state. Blot-drying technique helps to keep wetness of dentin substrate in an appropriate state for better adhesive penetration and higher bond strengths are obtained. The results of the present investigation indicate that higher bond strength values are obtained when blot-drying method is used with all three TE adhesive systems, however statistically different results were only observed in XP group. This is in correlation with the previous studies in which blot-drying method yielded higher bond strengths [6, 14]. In one these studies, air-drying time was 1 s and 10 s [14]; contrarily, in our study it was 5 s. It is quite possible that 1 s air-drying would yield a moist surface when compared with 10 s air-drying.

Air-drying method that we used in this study is also a wet bonding technique, since we air-dried the dentin ensuring that surface was visibly moist. However, this method showed lower bond strengths when compared with blot-drying method. A study compared blot-drying and 3 s gentle air-drying and showed better results with blot-drying method [13]. In that study, authors tested an acetone based, a water based and a water/ethanol based adhesive and found that blot-drying performed the best. Another study compared XP Bond with acetone or ethanol containing TE adhesives, and found better bond strength values for blot-drying [15]. Blot-drying method is more controllable application for leaving dentin surface moist and ensures removal of excess water from surface unvaryingly. On the other hand, air-drying method is more complex; time, angle, position and pressure dependent technique thus final moisture of dentin surface may not be consistent [12]. In our study, air-drying was performed from a distance of 10 cm. This distance was chosen with reference to a study that found 10 cm air-drying distance performed better than 1 cm air-drying distance [12].

Solvents in total-etch systems should promote penetration of the monomers in the collagen network and be capable of re-expanding the collapsed collagen network if dentin is air-dried [6, 16, 17]. Ethanol, water and acetone are mainly used solvents in dental adhesives. Some characteristics of a solvent may have important factors on dentin bond strength. These are hydrogen bonding capacity, vapor pressure, boiling point, dielectric constant and dipole moment [7]. In dried dentin, the hydrogen bonding capacity of solvent is important for re-expanding the shrunken collagen network [10]. In order to break stabilizing hydrogen bonds and other forces that keep the collagen in shrunken state; solvents should have high affinity to form hydrogen bonds.

Water has high dielectric constant and capable of breaking the hydrogen bonds between collagen fibers as a solvent but due to its low vapor pressure it is difficult to remove it from tooth after application [18, 19]. Ethanol has higher vapor pressure when compared with water and allows better evaporation by air-drying. Ethanol based solvents also contain water as co-solvent and this mixture results in a better evaporation than pure water [20]. According to some studies; ethanol has a stiffening effect on demineralized collagen thus maintains interfibrillar spaces in collagen network [21, 22]. When compared with water and ethanol higher vapor pressure of acetone allows better evaporation of solvent from the applied adhesive. Because of its high dipole moment and high evaporative ability acetone has a water chasing effect that helps removing excess water from tooth surface. Due to low hydrogen bonding capacity of acetone, re-expansion of shrunken collagen is not possible [10]. Therefore acetone containing adhesives should be applied with wet bonding technique [23].

Although acetone based system showed higher SBS values than ethanol based adhesive system no statistically different results were observed. Solvent type of adhesive systems have effect on the bond strength [24]. Some studies showed that ethanol based systems had higher bond strengths than acetone based systems [25–28]. On the other hand, several studies showed that acetone based systems provide similar or higher bond strength values to dentin than ethanol based systems with wet bonding techniques as they have a very good water removing capacity [11, 29–32]. Although there was no significant difference in between; when applied with wet bonding technique, acetone based system showed higher SBS values than ethanol based system in our study.

A study hypothesized that chemical interaction with XP bond and demineralized dentin occurred by formation of calcium phosphate complexes derived from mineral apatite in dentin and phosphate esters in the adhesive [33]. According to its manufacturer; the use of dipentaerythritol pentaacrylate monophosphate (PENTA) and butan-1,2,3,4-tetracarboxylic acid di-2-hydroxyethylmethacrylate ester (TCB) resin as adhesion promoters in the XP bond promotes chemical interaction between the monomers and tooth substance and ensures high bond strength to tooth substance (XP BOND. Konstanz, Germany: Dentsply DeTrey, 2006). In the present study, statistically significant differences were observed in different solvent based adhesive systems; and tertiary butanol based groups showed higher SBS values in both drying methods. Higher bond strength values of XP group might be explained by its chemical composition; because of the tertiary group, tertiary butanol is not able to chemically react with the resins in the same way as ethanol and provides an increase in resin content [34]. This increase in resin content provides thicker and denser polymer matrix after polymerization and this may result in higher bond strengths. XP-blot produced significantly higher SBS than XP-air. This result may be attributed to better moisture spectrum achieved through blot-drying method.

Dental adhesive systems are composed of many different ingredients and each of them have an effect on their performances [7]. However, resin components and solvent play a more important role. In this study, different TE adhesives with three different solvents were evaluated. Tertiary butanol containing adhesive showed higher bond strength values when compared with acetone or ethanol containing adhesives. An in vitro study found that a tertiary butanol containing two-step TE adhesive (XP Bond) sealed the dentin tubules better than acetone or ethanol containing systems [34]. Six-month clinical performance of tertiary butanol based TE adhesive was found to be equal when compared with ethanol/water based TE adhesive [35].

Adhesive systems evaluated in this study, not only differ in terms of solvent type, but also in terms of monomer content. Therefore, results we obtained in our study might also have been influenced by presence of other ingredients. However, it is well known that, solvents play an important role in promoting good penetration of the monomers in the collagen network of the demineralized dentin in TE adhesives [16], thus might have a profound effect on bond strength performance. Care should be taken when interpreting the results of the studies; since adhesive formulations are complex and contain many different ingredients. Initiator systems, filler, inhibitors and some specific ingredients may also affect performance of adhesive systems. In this study, we only assessed the effect of solvent and drying method on bond strength of TE adhesives. Bond strength is not the only consideration to evaluate their performance. Microscopic and detailed assessment of the bonding interface might give useful data and help enlighten the interactions in these interfaces. Therefore, further studies, utilizing more advanced analysis techniques should be performed to assess the effect of solvent type on adhesive performance.

## Conclusions

Present study suggests that solvent type and drying methods might have an effect on bond strengths of one-step total-etch adhesives to dentin. Blot drying method and tertiary butanol based adhesive shows higher bond-strengths values. Further research is necessary to determine in vivo and in vitro performance of tertiary butanol based total-etch adhesive systems.

## Abbreviations

Bis-GMA, bisphenol A diglycidyl methacrylate; BPDM, biphenyl dimethacrylate or 4,40-dimethacryloyloxyethyloxycarbonylbiphenyl-3,30-dicarboxylic acid; GPDM, glycerol phosphate dimethacrylate; HEMA, 2-hydroxyethyl methacrylate; PENTA, dipentaerythritol pentaacrylate monophosphate; TCB, butan-1,2,3,4-tetracarboxylic acid di-2-hydroxyethylmethacrylate ester; TEGDMA, triethylene glycol dimethacrylate; UDMA, urethane dimethacrylate or 1,6-di(methacryloyloxyethylcarbamoyl)-3,30,5-trimethylhexaan
